# Cardiac Tamponade Due to Pericardial Effusion Following Peripherally Inserted Central Catheter: A Single-Institution Case Series

**DOI:** 10.7759/cureus.56403

**Published:** 2024-03-18

**Authors:** Ha T Trinh, Thien T Nguyen, Tinh T Nguyen

**Affiliations:** 1 Neonatal Intensive Care Unit, Children’s Hospital 2, Ho Chi Minh City, VNM; 2 Pediatrics, University of Medicine and Pharmacy, Ho Chi Minh City, VNM; 3 Neonatology, University Medical Center, Ho Chi Minh City, VNM

**Keywords:** peripherally inserted central catheters, shock, point-of-care ultrasound, pericardial effusion, cardiac tamponade

## Abstract

Introduction: Although the use of peripherally inserted central catheters (PICCs) has many advantages, misplacement can lead to serious life-threatening complications such as pericardial effusion (PCE) and cardiac tamponade (CT). This report aims to describe four cases of CT resulting from misplaced PICC, which were successfully managed.

Methods: Retrospective analysis of neonates who required PICC insertion and had PCE leading to CT in the Neonatal Intensive Care Unit (NICU) at The Children’s Hospital 2, Ho Chi Minh City, Vietnam, during the year 2022.

Results: Four cases involved preterm infants at 28-30 weeks gestational age, weighing between 900-1,500 grams. The PCE/CT developed between 3 and 24 days following PICC insertion. The abrupt onset with clinical manifestations that showed hemodynamic instability included sudden deterioration, lethargy, apnea, bradycardia, pale skin, and cardiovascular collapse. We use cardiac point of care ultrasound (POCUS) to assess the condition of these patients and guide the pericardiocentesis procedure. The analysis of the aspirated fluid used for PCE/CT treatment is consistent with the component of parenteral nutrition. No deaths were encountered.

Conclusion: Neonates presenting sudden deterioration following PICC insertion should undergo POCUS to prompt identifying PCE/CT. Timely diagnosis via POCUS, prompt pericardiocentesis, and prevention of misplaced PICC-associated serious complications are crucial. Monitoring of the PICC position twice a week is recommended to avoid life-threatening complications. Additionally, incorporating POCUS for identifying the tip of PICC rather than relying solely on X-ray should be considered in the current protocol.

## Introduction

Background

Peripherally inserted central catheter (PICC) lines are commonly used for parenteral nutrition in preterm infants. Although the use of PICC has many advantages, PICC misplacement can lead to severe complications such as pericardial effusion (PCE) and cardiac tamponade (CT). The incidence of PICC-associated CT ranges from 2.2% to 6.7%, with a mortality rate of up to 75% [1,2]. Our research was carried out at one of the largest pediatric hospitals in the region. All the newborns in our unit are out-born referred from Obstetrics and Gynecology or general hospitals. This study aims to describe cases of PCE/CT resulting from misplaced PICC, which were successfully managed.

Materials and methods

After receiving ethical approval from the Institutional Review Board in 2022, we performed a retrospective review of charts of neonates who required PICC insertion for parenteral nutrition. Neonates who developed PCE/CT secondary to PICC insertion were recruited. The medical records of these patients were collected and analyzed.

In our practice, we use a 1-Fr single-lumen Polyurethane catheter for PICC insertion. As per our protocol, the positions of the PICC tips were confirmed by X-rays. The catheter tip is correct when the tip aligns with the level of the fourth thoracic vertebra (T4) for upper limb PICC placement and at the level of the ninth thoracic vertebra (T9) for lower limb PICC placement. 

PICCs are secured in place using catheter dressings in accordance with the protocol established by our unit. These dressings are composed of sterile, adhesive materials and a tegaderm. Additionally, the catheters are marked at specific lengths to enable nurses to ascertain if there has been any displacement of the catheter, either inward or outward. The assigned nursing staff assesses the integrity of these dressings at every shift. It is our policy not to trim the catheters. The catheter is inserted to an estimated length suitable for the patient, with any excess length which is secured on the patient’s body.

According to the protocol established by our unit, the tip of the catheter is not confirmed on the radiograph, a bedside ultrasound was carried out to confirm its position. Hitachi Aloka Noblus ultrasound machine, 5 MHz probe with 4-chamber, 5-chamber, vertical parasternal, and horizontal parasternal views were used to investigate the cardiac images. Neonatologists who were trained in the point of care ultrasound (POCUS) carried out an initial ultrasound. If PCE and CT were detected, it would be confirmed by a cardiologist. A cardiologist and a cardiac surgeon decide the appropriate treatment for the individual.

## Case presentation

In 2022, there were 1,021 newborns admitted to our Neonatal Intensive Care Unit (NICU); 207 infants of these were received a PICC. Our NICU encountered six cases (2.9%) of PICC-associated PCE, and of these, four cases (1.9%) of PICC-associated PCE/CT due to migrated PICC tip subsequently. Below is a detailed description of each case.

Case 1

**Figure 1:**
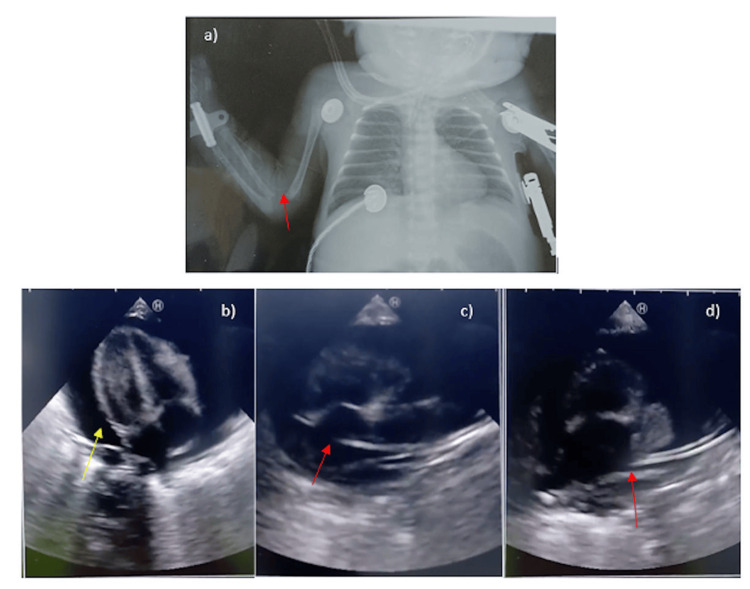
Echocardiogram and chest radiograph of Case 1 a) Supine chest radiograph following PICC insertion, the catheter tip was not visualized; b) PCE; c) PICC tip positioned within the right atrium; d) PICC tip located at the junction of the superior vena cava and the right atrium after the catheter was withdrawn. PICC: Peripherally inserted central catheter; PCE: Pericardial effusion

A 28-week gestational age male neonate with a birth weight of 900 g was admitted to the NICU. The infant was suspected to have intestinal obstruction and was initially supported with nasal continuous positive airway pressure (NCPAP), then leading to an ileostomy at 51 hours of age. A PICC was inserted through the right basilic vein at the elbow with a length of 13.5 cm at 22 days old. Six days post-PICC placement, the patient suddenly presented signs of lethargy, irritability, and pallor, leading to the subsequent need for invasive mechanical ventilation. An arterial blood gas indicated severe metabolic acidosis. A neonatologist-conducted point-of-care ultrasound showed significant PCE with the PICC tip in the right atrium (Figure 1 and Video 1). The bedside ultrasound was used to guide the withdrawal and correction of the PICC line. A pericardiectomy was performed by an on-call cardiac surgeon consultant to address the PCE/CT. The fluid analysis was consistent with parenteral nutrition (Table 1). On post-operation day 5, ultrasound showed resolution of the PCE. The patient had marked improvement in clinical status. Unfortunately, enteritis was progressive; the patient could not tolerate enteral feeding for a month, experienced thrombocytopenia, and was undergoing treatment with prolonged broad-spectrum antibiotics. He subsequently developed septic shock and died a week later. 

**Video 1:** Case 1-Echocardiogram showing CT A large circumferential PCE compressing the heart is visualized by transthoracic echocardiography (subcostal 4-chamber view). The signs of acute CT included (i) a large PCE, (ii) swinging motion of the heart, and (iii) the systolic collapse of the right atrium. PCE: Pericardial effusion; CT: Cardiac tamponade

**Table 1:** Summary of the characteristics, clinical features, complications, treatments, and outcomes of the four cases PICC: Peripherally inserted central venous catheter; CT: Cardiac tamponade; PCE: Pericardial effusion; RBCs: Red blood cells; WBCs: White blood cells; NCPAP: Nasal continuous positive airway pressure

No.	Gestational Age (wks)	Sex	Birth weight (g)	Insertion site	Age at insertion – complication (days)	Type of infusate	Clinical features	Echocardiography	Treatment	Outcome
1	28	Male	900	Right basilic vein	22 – 28	Lipid 20%, Amino acid 6.5%, Glucose, Minerals; Glucose concentration: 7.9%; Osmolarity: 780 mOsm/L	Sudden lethargy, irritability, pallor, and required mechanical ventilation	A large PCE, swinging motion of the heart, collapse of the right atrium	The bedside ultrasound was used to guide the withdrawal and correction of the PICC line. Pericardiectomy was done.	Dead at 34 days old due to septic shock
2	28	Female	900	Right basilic vein	55 – 79	Amino acid 6.5%, Glucose, Minerals (without lipids); Glucose concentration: 8.4%; Osmolarity: 803 mOsm/L	Sudden lethargy, apnea, bradycardia, pallor, and required intubation	A large PCE, swinging motion of the heart, collapse of the right atrium	The PICC was removed. 20 mL of yellow fluid was aspirated from the pericardial space.	Survived, discharged at 144 days old
3	30	Female	1,460	Right basilic vein	5 – 8	Lipid 20%, Amino acid 6.5%, Glucose, Minerals; Glucose concentration: 8%; Osmolarity: 860 mOsm/L	Sudden irritability, weak pulses, faint heart sounds, bradycardia	Collapse of the right atrium	Cardiopulmonary resuscitation. Vasoactive and inotropic medications were administered. The PICC was removed.	Survived, discharged at 41 days old
4	28	Female	1,200	Right superficial temporal vein	10 – 26	Amino acid 6.5%, Glucose, Minerals (without lipids); Glucose concentration: 5.5%; Osmolarity: 610 mOsm/L	Respiratory fatigue, required NCPAP support	A large PCE, swinging motion of the heart, collapse of the right atrium	Pericardiectomy was done.	Survived, discharged at 107 days old

Case 2

A 28-week gestational age female neonate weighing 900 g was admitted to the NICU. After birth, she was suspected to have visceral perforation and was supported with invasive mechanical ventilation. She was admitted to our hospital at six days old and underwent surgery for segmental bowel resection, ileostomy, and parenteral nutrition at seven days old. 

**Figure 2:**
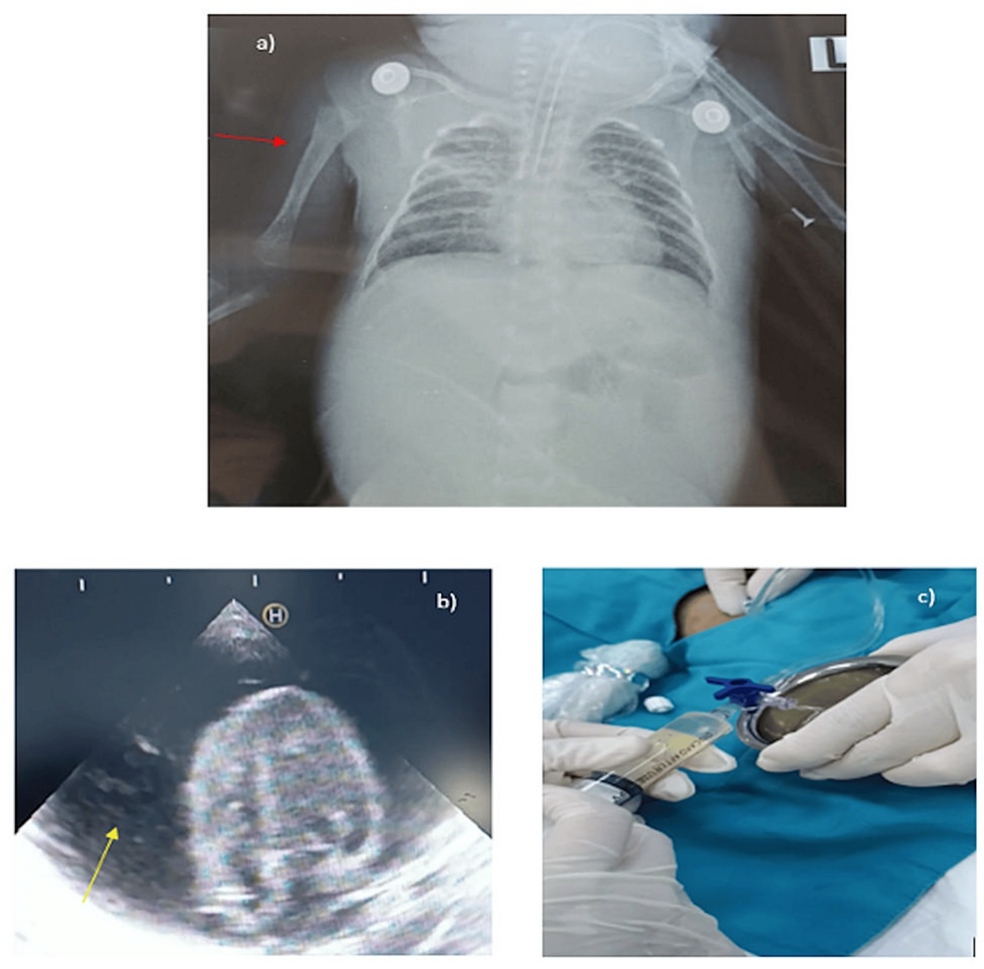
Echocardiogram, chest radiograph, and pericardial fluid of Case 2 a) Chest X-ray performed in a supine position to confirm the PICC position but unable to reveal catheter tip position; b) PCE; c) Pericardiocentesis PICC: Peripherally inserted central catheter; PCE: Pericardial effusion

A PICC was inserted through the right basilic vein at an elbow at a length of 13 cm on 55 days of life. 24 days after PICC placement, while on NCPAP, the infant suddenly presented lethargy, apnea, bradycardia, pallor, and required intubation. The patient had severe metabolic acidosis. POCUS by a neonatologist revealed significant PCE. The PICC was removed, and under the guidance of POCUS, 20 mL of light yellowish fluid was aspirated from the pericardial space (Figure 2 and Video 2). The clinical status quickly improved. Ultrasound performed after pericardiocentesis showed no PCE. The fluid analysis was consistent with parenteral nutrition (Table 1). She was weaned off ventilation and subsequently discharged on 144 days of life.

**Video 2:** Case 2-Echocardiogram showing CT A large circumferential PCE compressing the heart is visualized by transthoracic echocardiography (subcostal 4-chamber view). The signs of acute CT included (i) a large PCE, (ii) swinging motion of the heart, and (iii) the systolic collapse of the right atrium. PCE: Pericardial effusion; CT: Cardiac tamponade

Case 3

A 30-week gestational age female birth weight of 1,460 g was admitted to the NICU. After birth, the infant experienced respiratory failure, requiring invasive mechanical ventilation, and was subsequently referred to our hospital at 19 hours old. 

**Figure 3:**
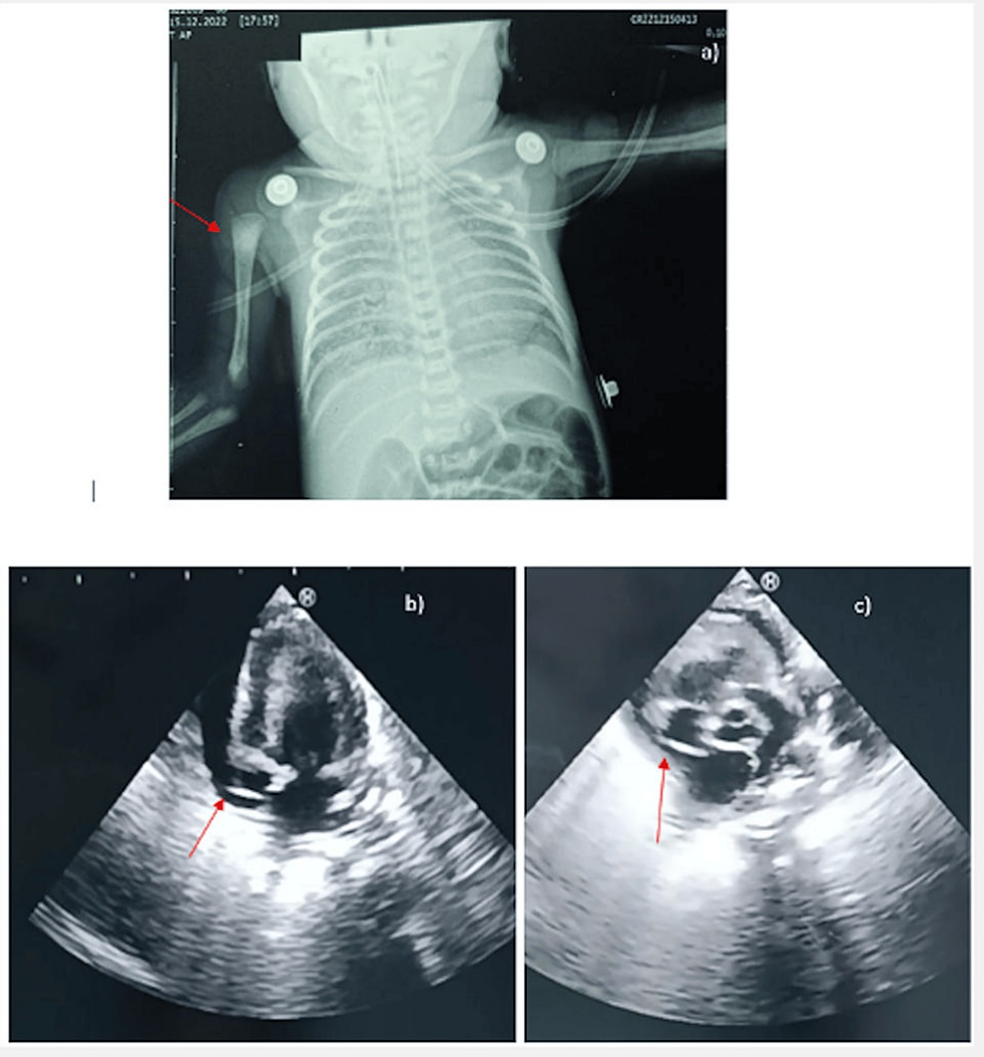
Echocardiogram and chest radiograph of Case 3 a) Chest X-ray performed in a supine position to confirm the PICC position but unable to reveal catheter tip position; b) PCE with the PICC tip within the right atrium; c) PICC tip within located the right atrium PICC: Peripherally inserted central catheter; PCE: Pericardial effusion

Upon admission to our unit, a PICC was placed in the basilic vein of her right elbow at a length of 14 cm at five days old. Three days after PICC placement, she presented sudden irritability, weak pulses, faint heart sounds, and bradycardia. Cardiopulmonary resuscitation was initiated, vasoactive and inotropic medications were administered, and an urgent blood gas revealed severe metabolic acidosis. Urgent cardiac ultrasound showed PCE measuring 5-7 mm in diameter, in uniform appearance with the systolic collapse of the right atrium (Figure 3 and Video 3). The PICC was removed. Ultrasound-assisted aspiration retrieved 5 mL of yellow fluid, reducing residual pericardial fluid to about 2 mm in diameter and resolving CT. Vasoactive and inotropic medications were gradually reduced, and clinical status was improved. The biochemical analysis of the pericardial fluid was consistent with the composition of parenteral nutrition (Table 2). A follow-up ultrasound three days later showed no residual effusion, and the neonate was successfully weaned off ventilation and subsequently discharged on 41 days of life.

**Video 3:** Case 3-Echocardiogram showing PCE Case 3 did not have as much effusion on ultrasound as the other three cases, but there was systolic collapse of the right atrium. Still, the velocity of effusion, which is the duration of PCE from the time of PICC insertion, was very short compared to the other three cases, so the heart may not have enough time to adapt, and the cardiac function still collapsed. This phenomenon is also described in the literature, the clinical presentation and the patient’s hemodynamics substantially hang on the velocity of fluid accumulation and influence the decision to intervene. When the PCE accumulates rapidly or presents in a tamponade, pending tamponade, or is causing significant respiratory or hemodynamic instability, pericardiocentesis is usually performed. The baby was on a ventilator and suddenly had a serious cardiovascular collapse. Cardiopulmonary resuscitation was initiated, vasoactive and inotropic medications were administered, and an urgent blood gas revealed severe metabolic acidosis. PCE: Pericardial effusion; PICC: Peripherally inserted central catheter

**Table 2:** Summary of pericardial fluid and arterial blood gas characteristics of the four cases RBC: Red blood cell; WBC: White blood cell; pCO_2_: Partial pressure of carbon dioxide; pO_2_: Partial pressure of oxygen; BE: Base excess; HCO_3_^-^: Bicarbonate

	Parameters	Case 1		Case 2		Case 3		Case 4		Reference range
Pericardial fluid biochemistry	Tryglyceride	0.39		0		Not available		0		0 mmol/L
	Glucose	17.4		44.4				6.2		0 mmol/L
	Protein	1.7		1.26				1.79		0 g/L
	RBCs	40		0				700		0 cells/mm^3^
	WBCs	111		164				75		0 cells/mm^3^
Arterial blood gas		Before treatment	9 hours after treatment	Before treatment	4 hours after treatment	Before treatment	3 hours after treatment	Before treatment	6 hours after treatment	
											pH	6.94	7.23	6.98	7.42	7.02	7.29	Not available	7.26	7.35 – 7.45
											pCO_2_	42	36.7	58	23.6	32	36		55	35 – 45 mmHg
	pO_2_	54	107	94	52	64	86	77	50 – 80 mmHg									
										BE	-22	-11	-19	-8	-21	-8	-2		(-2) – (+2) mmol/L
	HCO_3_^-^	9.1	15.6	13	14.7	8.4	17.7	25.3	20 – 24 mmol/L										

Case 4

A 28-week gestational age female neonate with a birth weight of 1,200 g was referred to our hospital due to a suspected tracheoesophageal fistula at 26 days old. In our neonatal unit, her respiratory condition improved. She was on room air, and tracheoesophageal fistula was ruled out. A PICC was inserted through the right superficial temporal vein at a length of 12.5 cm. 

**Figure 4:**
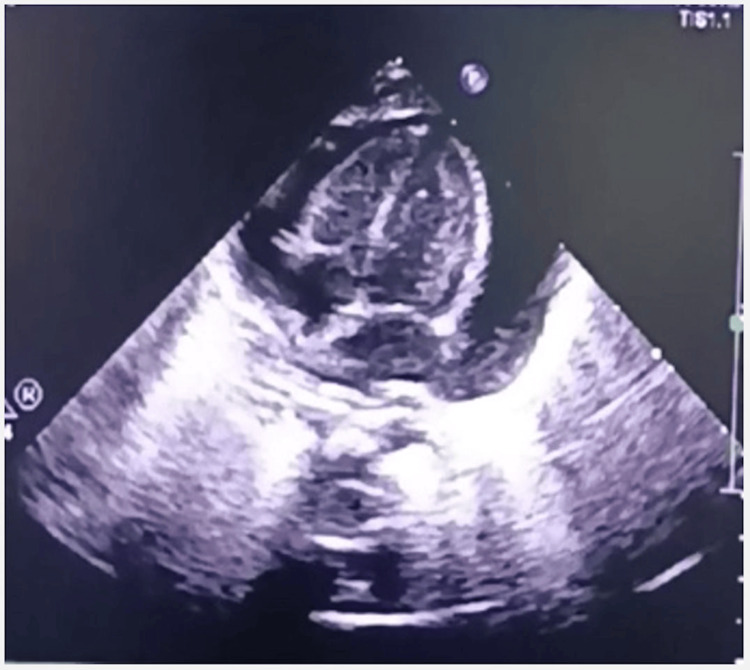
Echocardiogram and chest radiograph of Case 4 Echocardiogram showing PCE with CT PCE: Pericardial effusion; CT: Cardiac tamponade

On the 10th day after PICC insertion, which was also the scheduled day for PICC’s removal, she developed respiratory failure, requiring NCPAP support. A cardiac ultrasound showed significant PCE. The baby was intubated for mechanical ventilation and transferred to the operation room for surgical drainage, and the PICC was removed (Figure 4 and Video 4). On the third day of post-surgical drainage, echocardiography showed PCE resolution. The infant was successfully weaned off mechanical ventilation, and the clinical status was improved. The fluid analysis was consistent with parenteral nutrition and subsequently discharged at 107 days of life (Table 1).

**Video 4:** Case 4-Echocardiogram showing CT A large circumferential PCE compressing the heart is visualized by transthoracic echocardiography (subcostal 4-chamber view). The signs of acute CT included (i) a large PCE, (ii) swinging motion of the heart, and (iii) the systolic collapse of the right atrium. PCE: Pericardial effusion; CT: Cardiac tamponade

## Discussion

The incidence of CT related to PICC ranges from 2.2% to 6.7% [1]. Although it is uncommon, early suspicion and detection are crucial due to high mortality rates of up to 75% without pericardiocentesis and 8% with pericardiocentesis [2].

According to Warren's autopsy study, which presented the gross and histopathologic findings in five neonates receiving continuous total parenteral nutrition via central venous catheters (CVCs), who suddenly and unexpectedly died from PCE/CT. These cases were unresponsive to aggressive resuscitation and died soon. Autopsy findings revealed PCE and CT. The author suggested several mechanisms of pericardial damage caused by CVC, including (i) myocardial damage and micro-perforation associated with mechanical right atrial injury by direct right atrial contact with the catheter tip; (ii) permeation of parenteral nutrition components with high osmotic pressures, through the right atrial wall without macroscopic or microscopic cardiac injuries, but demonstrated by interstitial edema and the endomyocardial microvascular dilatation [3].

Clinical manifestations of acute CT observed in our study included sudden clinical deterioration, apnea, bradycardia, pallor or circulatory collapse, and metabolic acidosis. All infants underwent urgent neonatologist-conducted cardiac POCUS and echocardiography-guided pericardiocentesis or surgical pericardiectomy based on local expertise, leading to successful outcomes. Our clinical presentations and the analysis of pericardial fluid are consistent with those of other studies [1,4-6].

In our study, three cases underwent PICC removal, while one had the catheter partially withdrawn out of the right atrium, and it remained in a proper position. We did not remove this PICC because it was determined that further attempts at central venous access would be technically difficult, and echocardiography showed the correct tip position of the catheter. Similarly, Nowlen reported 21 out of 54 cases with PCE in which catheters were partially withdrawn but remained in use. It was concluded that complete catheter removal was unnecessary, but close monitoring was essential to prevent recurrent PCE [2].

To prevent PICC-associated PCE/CT, accurate catheter tip positioning is crucial. The Manchester report, in 2001, suggested placing the tip of CVCs should remain outside the cardiac silhouette but still within the vena cava on chest X-rays in premature infants [2,7]. In 2002, the US Food and Drug Administration (FDA) recommended avoiding placement of the tip of the catheter into the right atrium and minimizing an infant's movement to prevent catheter migration into the right atrium [8]. Recommendations included placing the catheter tip outside the cardiac silhouette, preferably in the upper vena cava or lower vena cava connected to the right atrium, 0.5-1 cm for preterm infants and 1-2 cm for term infants above the diaphragm on chest X-rays [2,9]. According to the recommendations of the National Health Service (NHS) in the United Kingdom, the catheter tip of a PICC should be positioned above the level of the fourth thoracic vertebra (T4) for upper limb PICC placement and above the level of the ninth thoracic vertebra (T9) for lower limb PICC placement [10]. Several studies have indicated that the catheter tips can move toward the heart with any movement of the related parts, such as the head or limbs, increasing the risk of PCE [2,11,12].

In our study, the duration from PICC insertion to the detection of PCE and acute CT ranged from three days to a maximum of 24 days. This variation can be attributed to the movement characteristics of the PICC, which pose a risk of PCE. Before and immediately after the circulatory collapse, laboratory tests were conducted on the four infants. The results indicated no ongoing infections or pneumothorax; the only finding was severe metabolic acidosis. Echocardiography results, as detailed in the attached videos, verified these findings. Following the removal of pericardial fluid through pericardiocentesis or pericardiectomy, the patients experienced a significant improvement in their clinical conditions, particularly respiratory distress and hypotension/shock. There was also a significant improvement in metabolic acidosis and a complete resolution of PCE. Case 3 did not have as much effusion on ultrasound as the other three cases. Still, the velocity of effusion, which is the duration of PCE from the time of PICC insertion, was very short compared to the other three cases, so the heart may not have had enough time to adapt, and the cardiac function still collapsed. This phenomenon is also described in the literature, the clinical presentation and the patient's hemodynamics substantially hang on the velocity of fluid accumulation and influence the decision to intervene. When the PCE accumulates rapidly or presents as tamponade, pending tamponade, or is causing significant respiratory or hemodynamic instability, pericardiocentesis is usually performed [13]. In the last two years, we have implemented POCUS. It is possible that prior to this period, cases of acute CT leading to sudden death might have been missed.

Interestingly, this implementation has not led to an increase in the incidence of PICC-associated PCE or pericarditis. As a result, we recommend the use of POCUS for neonates in the NICU showing signs of sudden clinical deterioration. This recommendation is partly due to the potential for catheter migration over time. Therefore, to mitigate this risk, we recommend regular catheter inspections and checks, ensuring they are secured at the insertion site and have close monitoring by nursing staff. As there is currently no standardized recommendation for regular PICC tip position monitoring, we suggest checking the PICC position twice a week to prevent complications related to the PICC.

Anterior-posterior chest X-rays are frequently used in neonatal units. However, a chest X-ray cannot correctly determine the catheter tip position, and it increases the risk of radiation exposure for both infants and healthcare personnel in the neonatal unit. In our study, although three cases underwent chest X-ray examination, the catheter tip position was not clearly visible. Additionally, the Sneath study suggested that ultrasound can identify the malposition catheter's tip even when chest X-rays appear normal [9]. Numerous authors recognize ultrasound as a superior method for evaluating catheter tip position compared to chest X-rays. Even catheters as small as 1 French can be clearly visualized using ultrasound, which is safe and accurate without causing radiation exposure. Neonatologists can do it beside. However, they needed to be trained [14-16]

In Khoo's study, all three preterm infants who experienced PCE/CT used PICC-1F polyurethane catheters [4]. Additionally, Wang et al. conducted a meta-analysis of risk factors for catheter-related PCE in neonates showed polyurethane catheters led to significantly more neonatal PCE than silicone counterparts (P< .01) [1]. Catheters with a guidewire resulted in higher mortality rates compared to umbilical venous catheters (UVC) and PICCs (P < .05). Low birth weight infants tended to develop catheter-related PCE leading to higher mortality rates. However, the differences were not statistically significant (P> .05). The risk of catheter-related PCE was higher in PICC inserted via the upper limbs compared to the lower limbs. To minimize movement, in many NICUs, the neonatologists choose the lower limbs for PICC placement [1]. In our study, all infants used 1F catheters made of polyurethane material, weighed less than 1,500 grams, and had catheters positioned in the upper limbs or head.

In our unit, due to the availability of bedside ultrasound machines and echocardiography conducted by experienced neonatologists who have received training in neonatal cardiac ultrasound, all cases were quickly diagnosed, resulting in successful treatment outcomes. Similarly, in recent times, an increasing number of neonatologists are focusing on implementing neonatal POCUS. POCUS has become an indispensable part of clinical examination, facilitating better diagnosis, treatment, and various procedures, ultimately benefiting neonatal care [17]. POCUS is rapidly advancing, and many guidelines for neonatologist-performed echocardiography have been published recently [18,19].

Strengths

To our knowledge, this report is the largest case series from a single center within a year, with the cases presenting significant similarities. It highlights the pivotal role of neonatologists in utilizing POCUS for early diagnosis and timely management of PCE/CT. The findings recommend that neonatologists should consider PCE/CT and carry out POCUS in infants presenting sudden deterioration following PICC placement, aiming for early detection of PCE/CT.

Limitations

There is no direct evidence to definitively confirm that this PCE/CT is attributed to the PICC, as we did not carry out a cardiac pathology examination. However, all the cases were successfully managed through pericardiocentesis or pericardiectomy, accompanied by PICC removal repositioning. The analysis of pericardial fluid in three out of four cases was consistent with intravenous nutrition, suggesting an association with the PICC.

## Conclusions

Neonates with a sudden unexplained deterioration after the placement of a PICC should undergo emergent bedside ultrasound to investigate PCE/CT. Prompt identification and timely pericardiocentesis can be lifesaving. Bedside ultrasound may play a crucial role in early detection and prevention of complications associated with misplaced PICC’s tip. Regular monitoring of the PICC position twice a week was recommended to mitigate complications. Additionally, incorporating POCUS for identifying the tip of PICC instead of solely on X-ray should be considered in the current protocol.
